# Recent advancement of sonogenetics: A promising noninvasive cellular manipulation by ultrasound

**DOI:** 10.1016/j.gendis.2023.101112

**Published:** 2023-09-15

**Authors:** Jin Tang, Mingxuan Feng, Dong Wang, Liang Zhang, Ke Yang

**Affiliations:** a Pediatric Research Institute, Children's Hospital of Chongqing Medical University, National Clinical Research Center for Child Health and Disorders, Ministry of Education Key Laboratory of Child Development and Disorders, China International Science and Technology Cooperation Base of Child Development and Critical Disorders, Chongqing Engineering Research Center of Stem Cell Therapy, Chongqing 400014, China; b Department of Ultrasound, The First Affiliated Hospital of Chongqing Medical University, Chongqing 400016, China; c Tongji Medical College, Huazhong University of Science and Technology, Wuhan, Hubei 430074, China

**Keywords:** Mechanosensitive channel, Neuromodulation, Sonogenetics, Ultrasound, Ultrasound-sensitive protein

## Abstract

Recent advancements in biomedical research have underscored the importance of noninvasive cellular manipulation techniques. Sonogenetics, a method that uses genetic engineering to produce ultrasound-sensitive proteins in target cells, is gaining prominence along with optogenetics, electrogenetics, and magnetogenetics. Upon stimulation with ultrasound, these proteins trigger a cascade of cellular activities and functions. Unlike traditional ultrasound modalities, sonogenetics offers enhanced spatial selectivity, improving precision and safety in disease treatment. This technology broadens the scope of non-surgical interventions across a wide range of clinical research and therapeutic applications, including neuromodulation, oncologic treatments, stem cell therapy, and beyond. Although current literature predominantly emphasizes ultrasonic neuromodulation, this review offers a comprehensive exploration of sonogenetics. We discuss ultrasound properties, the specific ultrasound-sensitive proteins employed in sonogenetics, and the technique's potential in managing conditions such as neurological disorders, cancer, and ophthalmic diseases, and in stem cell therapies. Our objective is to stimulate fresh perspectives for further research in this promising field.

## Introduction

Ultrasound is extensively employed in medicine for diagnostic and therapeutic purposes. As a form of mechanical energy, ultrasound waves can be efficiently delivered deep into soft tissues, up to several tens of centimeters, while preserving spatial and temporal coherence.^1^ Ultrasonic imaging has gained wide acceptance as a diagnostic modality, offering real-time, noninvasive imaging at low cost with high portability and safety. Moreover, focused ultrasound (FUS) presents a promising treatment approach for certain conditions, including noninvasive ablation of cancer and neuromodulation, by focusing energy deep into tissues. Furthermore, ultrasound-mediated biological effects have demonstrated potential in healing bone fractures, enhancing soft-tissue regeneration, and improving drug delivery.^2^

In recent decades, advancements in ultrasound technology have highlighted its potential in cellular manipulation. Specifically, it can transmit sound pressure waves that can effectively stimulate or inhibit neuronal activity in both peripheral and central nerves, thereby facilitating electrically evoked response^3^, ^4^, ^5^ Consequently, ultrasonic neuromodulation emerges as a viable treatment for neurological disorders, such as Parkinson's disease, Alzheimer's disease, epilepsy, and chronic pain.^6^, ^7^, ^8^, ^9^, ^10^, ^11^ Additionally, ultrasound has proven effective in guiding stem cell differentiation, proliferation, and migration, which results in enhanced muscle fiber and bone regeneration.^12^, ^13^, ^14^, ^15^, ^16^, ^17^ Ultrasound has also demonstrated potential in promoting functional recovery and nerve regeneration following nervous system injuries by stimulating neural stem cells, thereby expanding its potential applications in regenerative medicine.^18^^,^^19^

Despite the promising potential of ultrasound across a variety of applications, its precise use for cellular manipulation remains limited due to a lack of comprehensive understanding regarding its effects on cellular excitability. Consequently, achieving accurate targeting of cells in deep tissues with ultrasound energy poses a significant challenge. To tackle this, recent research efforts have been directed toward augmenting the acoustic sensitivity of specific cells using ultrasound-sensitive elements. This approach can enhance the ultrasound stimulation of certain cells and influence cellular behavior. Sonogenetics, a nascent technique, combines noninvasive ultrasonic modulation with genetically engineered cells that express ultrasound-sensitive proteins.^20^, ^21^, ^22^, ^23^, ^24^ The initial step in leveraging sonogenetics involves genetically engineering the desired cells to produce these ultrasound-sensitive proteins. Once this is achieved, specific ultrasound stimulation can instigate a sequence of cellular activities that have been pre-engineered. The applications of sonogenetics hold the potential to substantially improve the precision of cellular manipulation. The purpose of this review is to provide an extensive overview of the fundamental properties of ultrasound waves, and the role of ultrasound-sensitive proteins in the field of sonogenetics. Furthermore, this review emphasizes recent breakthroughs and potential applications of sonogenetics in several research areas, including neurology, oncology, ophthalmology, and stem cell research.

### Characteristics and biological effects of ultrasound

Ultrasonic waves are sound waves with frequencies exceeding the audible range of the human ear (>20,000 Hz) and thus surpassing the uppermost limit of human hearing. Unlike diagnostic imaging which utilizes ultrasound indirectly, therapeutic ultrasound relies on direct interactions between the sound field and tissue to elicit desired beneficial biological responses. Soft tissues propagate sound waves at approximately 1540 m/s, with reflection and scattering occurring where there is an acoustic impedance change.^25^ These ultrasonic waves are typified by high frequency, short wavelength, precise directionality, and robust penetrative capabilities.^26^ Ultrasound can traverse concave surfaces, focusing energy at a single point, a phenomenon termed FUS. The resulting bioeffects of FUS and non-FUS differ, contingent upon various parameters like intensity, frequency, and stimulation site.^27^^,^^28^ A concentrated FUS beam can deliver equivalent energy with lesser frequency and duration than its non-focused counterpart. FUS boasts remarkable spatial resolution and tissue penetration, but accurately targeting specific cell types remains a challenge, even though the focal point of FUS can attain millimeter precision.^28^^,^^29^

**Figure 1:**
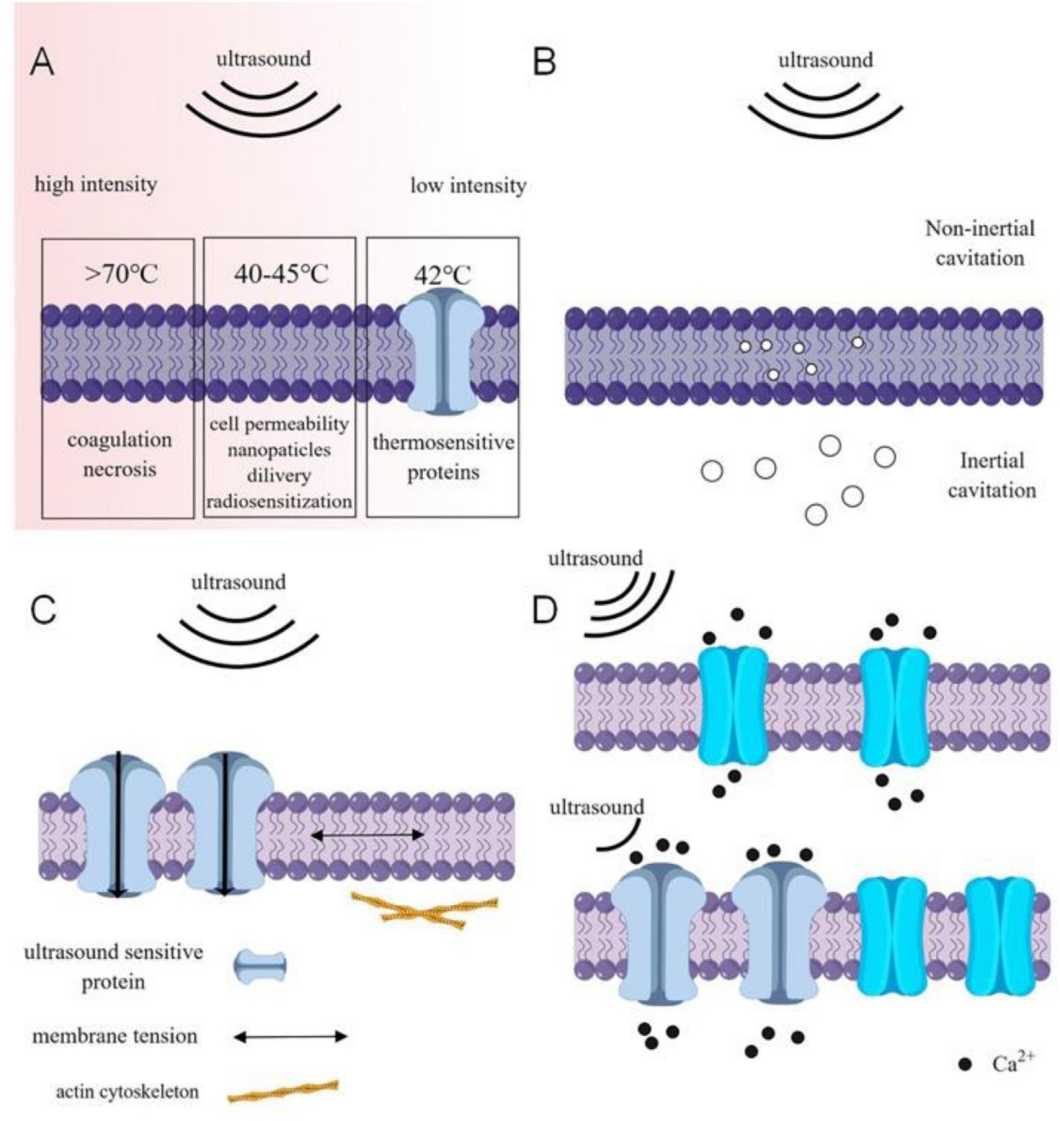
Schematic representation of the biological impacts of ultrasound. **(A)** Thermal effect: High-intensity focused ultrasound can increase tissue temperature beyond 70 °C, leading to coagulation necrosis; low-intensity focused ultrasound can also raise tissue temperature to 42 °C, activating thermosensitive proteins. **(B)** Cavitation: Acoustic pressure contributes to the formation and oscillation of gas bubbles, including inertial intra-membrane and non-inertial microbubbles. **(C)** Acoustic radiation force: The mechanical force exerted by ultrasound waves stimulates ultrasound-sensitive channels, affecting membrane tension and actin microfilament dynamics. **(D)** Sonogenetics: Ultrasound-sensitive proteins decrease the cell response threshold to ultrasound.

The biological effects of ultrasound mechanical vibrations on tissue can primarily be categorized into thermal and non-thermal impacts (Fig. 1). Thermal effects occur when ultrasound energy is absorbed, leading to a localized increase in tissue temperature, a process that hinges on ultrasound exposure parameters.^30^ High-intensity FUS can potentially ablate tumors by rapidly elevating tissue temperatures beyond 70 °C within milliseconds, resulting in irreversible coagulation necrosis.^31^ Moreover, ultrasound-induced temperature elevations of 40–45 °C can enhance cell permeability, improve nanoparticle transportation, and instigate radiosensitization.^32^ Brief applications of low-intensity FUS can trigger several thermosensitive proteins when the local tissue temperature reaches 42 °C.^33^

In terms of non-thermal effects, acoustic cavitation has been the focus of extensive research. This phenomenon involves the formation and oscillation of gas bubbles which can arise from pre-existing stable gas bodies or nuclei that stabilize within the liquid's impurity gaps.^28^ Upon a decrease in the liquid's pressure, the trapped gas expands, forming microbubbles.^34^ Non-inertial cavitation occurs when microbubbles oscillate around their equilibrium radius within the liquid, with the degree of response correlating to the acoustic field's frequency. This oscillation can generate heat, alter cell membrane potentials, and induce mechanical effects such as microstreaming and localized shear stresses. On the other hand, inertial cavitation is characterized by the rapid formation and collapse of microbubbles. In the presence of high-amplitude acoustic fields, bubbles may instantaneously expand and collapse, inflicting considerable damage to adjacent cells.

Acoustic radiation force describes the process whereby sound waves, upon encountering obstacles, translate sound energy into mechanical momentum. A low-intensity, periodic mechanical ultrasound wave propagates through a medium, inciting vibration and collisions. The bioeffects of acoustic radiation force are manifold, encompassing the modulation of cell proliferation and differentiation, the initiation of membrane channels, alteration of the cytoskeleton, and augmentation of protein expression.^35^, ^36^, ^37^ While the underpinning mechanism remains incompletely understood, recent investigations have shown that the mechanical force of ultrasonic radiation can stimulate ultrasound-sensitive molecules, thereby establishing a direct connection between ultrasound and cellular activities.^38^ Genetically engineering cells to express ultrasound-sensitive proteins engenders a specific reaction to ultrasound stimulation, an approach that is a burgeoning field with immense potential for enriching our understanding of cellular dynamics.

In order to circumvent potential tissue damage from ultrasound, acoustic radiation force and modest thermal effects are presently the primary biological mechanisms employed in sonogenetics. The selection of suitable ultrasonic effects and ultrasound-sensitive proteins is predicated on the characteristics of the tissue. The application of mild hyperthermia is fitting for neoplastic tissues or those insensitive to heat. However, in the case of central nervous tissue, a temperature increase may precipitate irreversible damage.

### Ultrasound-sensitive proteins

**Figure 2:**
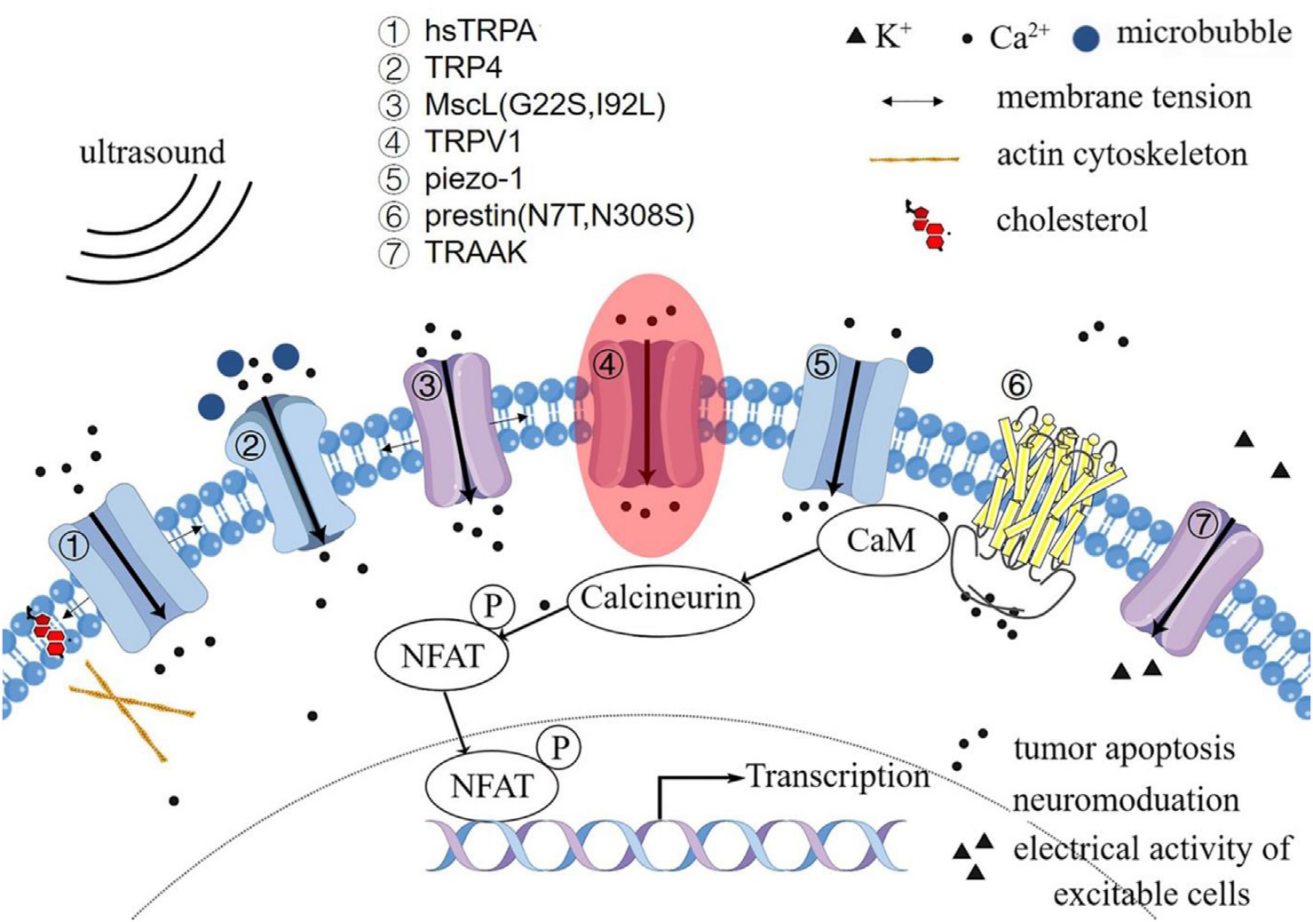
Ultrasound-sensitive proteins in sonogenetics.

The concept of sonogenetics was first introduced by Chalasani et al.^39^ One of the main challenges in advancing sonogenetics involves identifying a suitable ultrasound-sensitive protein to facilitate this technique (Fig. 2). The prevalent ultrasound-sensitive proteins identified to date are typically mechanosensitive channel proteins, which are expressed on the cellular membrane. These ion channel proteins rapidly transduce various forms of mechanical stimuli (such as shear forces, gravitational influence, tactile sensations, as well as internally derived osmotic pressure and membrane deformation) into electrical or biochemical signals. Consequently, these signals trigger deformation in the lipid bilayer, the ion channels' rotational movement, and the opening of channel pores, and ultimately lead to ion flux.^40^

### Mechanosensitive ion channels of large conductance (MscL)

The MscL, originating from the membrane surface of *Escherichia coli*, was the first cloned protein among the recognized mechanosensitive ion channels. This ubiquitous and highly conserved protein is prevalent in microorganisms and archaea, but homologs in humans and mammals have not been identified.^41^, ^42^, ^43^ Located in the inner membrane, MscL is a non-selective channel protein permitting the passage of water molecules, various ions (non-selective), and molecules with a mass of less than 10 KD.^44^^,^^45^ Structurally, MscL is a 3 nanoSiemen homopentameric mechanosensitive channel composed of 136 amino acid residues and an asymmetrical N2 terminus.^46^, ^47^, ^48^ Both the N-terminal and C-terminal of the polypeptide chain are situated on the cytoplasmic side of the cell membrane and are linked via the loop region. The MscL subunit comprises two transmembrane (TM) helix fragments, TM1 and TM2. Notably, studies have demonstrated that TM1 establishes the crux of a complex interplay between subunits within the transmembrane region. In contrast, TM2 predominantly interacts with membrane lipids and participates in the generation of membrane tension.^49^^,^^50^

Modifications to the amino acid residues of the MscL channel protein can facilitate its opening or closing in response to particular physical stimuli. For instance, the MscL G22C mutant within M1 transmembrane helices, when conjugated with light-responsive compounds, can reversibly modulate the channel pore's opening and closing state.^51^ Moreover, the pH-controlled alteration of the MscL G22C mutant, through association with a pH-regulating compound, modifies the hydrophobicity of the pore region by varying the pH level of the ambient environment.^52^^,^^53^ Furthermore, the combination of the MscL M42C protein mutant with the magnetic molecule CoFe_2_O_4_ lowers the mechanical threshold required for channel opening in the presence of a magnetic field.^54^

The MscL has the potential as a versatile tool for receiving diverse stimuli, given the comprehensive understanding of its foundational research, compact gene size, and expansive pore size. Additionally, MscL can be activated within the membrane by the effects of lipid bilayer tension, independent of any additional proteins or ligands.^55^ These observations suggest that MscL retains its mechanosensitivity when interfaced with vesicles, such as liposomes, and integrated into a lipid bilayer.^56^^,^^57^ The functional heterologous expression of engineered MscL can autonomously trigger neuron activity in response to mechanical stimulation, thereby paving the way for potential applications in sonogenetics to regulate neural function.^58^ A recent study has found that ultrasonic stimulation on dorsal striatum neurons expressing MscL-G22S increases motor activities both in anesthetic and freely moving mice and meanwhile activates the mesolimbic pathway to trigger dopamine release in the nucleus accumbens. Mutant MscL-G22S is more effective in sensitizing neurons to ultrasound than wild-type MscL.^59^ The phenomenon of ultrasound-induced channel opening, contingent on the presence of microbubbles and a structurally intact actin cytoskeleton, has been documented in retinal pigment epithelial cells expressing MscL.^60^ Importantly, primary cultured neurons expressing MscL mutants, like MscL-I92L or MscL-G22S, are more easily activated by low-pressure ultrasound pulses.^61^^,^^62^

### Transient receptor potential (TRP) channel proteins

TRP channel proteins constitute a family of non-selective cation channels possessing weak voltage sensitivity. They facilitate the inflow of cations, notably calcium and sodium. Found across all eukaryotes, TRP channels are notably prevalent within the central and peripheral nervous system in humans.^63^ The TRP channel family is composed of seven subfamilies: TRP canonical channels, TRP melastatin channels, TRP vanilloid channels, TRP melastatin channels, TRP mucolipin channels, TRP polycystin channels, and TRP ankyrin channels.^64^^,^^65^ Each protein in the family features a six-transmembrane structure (TM1–TM6), with both N-terminal and C-terminal ends located intracellularly. The non-selective cationic pore channel is formed by the conjunction of the fifth and sixth transmembrane structural domains. TRP channels can be activated by a multitude of stimuli, including temperature variations, pressure and volume changes, and certain substances such as cinnamon and camphor.^66^^,^^67^ Furthermore, mutations in TRP channels have been linked to a plethora of diseases, such as neurodegenerative disorders, various forms of cancer, glaucoma, and renal dysfunction.^68^, ^69^, ^70^, ^71^, ^72^, ^73^, ^74^, ^75^, ^76^

Transient receptor potential vanilloid 1 (TRPV1) is a non-selective cation channel, which can be activated by multiple stimuli, including heat, capsaicin, and an acidic pH. This channel has critical functions in nociception, inflammation, and thermoregulation. Moreover, TRPV1 is expressed in numerous brain regions, where it may regulate neuronal activity and synaptic plasticity. Initially identified as part of the thermosensitive transient receptor potential channel family, TRPV1 is activated at a temperature of 42 °C.^77^ Furthermore, previous research has reported that capsaicin and low extracellular pH (pH ≤ 5.9) can also activate TRPV1, leading to pain-associated behaviors in both animals and humans.^78^^,^^79^ Additionally, intracellular signaling pathway components, such as protein kinase C and phosphodiesterase C, can modulate TRPV1 activity. Importantly, the activation temperature for TRPV1 is slightly above the physiological body temperature, which allows the channel to close at normal body temperature. This feature facilitates rapid and safe stimulation while mitigating potential thermal effects.^77^^,^^80^ Recent research has shown that FUS-mediated sonogenetics can selectively activate calcium influx in neurons expressing the TRPV1 receptor. This activation has been observed to trigger locomotor behavior in freely moving mice by targeting either deep brain regions or superficial brain targets such as the motor cortex. These findings have significantly expanded the potential applications of TRPV1-mediated sonogenetics, now encompassing both superficial and deep brain targets.^33^^,^^81^

TRP-4 is a fascinating component of the TRP subfamily, comprising a pore region situated between TM5 and TM6, in addition to two intracellular amino acid sequences. Notably, TRP-4 has been identified to interact with calmodulin in the context of elevated Ca^2+^ concentrations. This distinctive characteristic underscores its role as a native store-operated Ca^2+^-permeable channel.^82^ Moreover, TRP-4 is pivotal in the process of mechanosensation and facilitates the conduction of ciliated mechanosensory neurons within *Caenorhabditis elegans*.^83^ Of particular interest, TRP-4 responds to ultrasound stimulation, thereby including calcium activity in *Caenorhabditis elegans* neurons at typical diagnostic ultrasound frequencies in the presence of microbubbles.^39^ It should be emphasized that TRP-4 lacks mammalian homologs, suggesting that its expression in mammalian brains might yield minimal additional effects.^83^ Mutations localized in the channel pore region of TRP-4 can potentially alter ion selectivity and sensitivity, thereby rendering it a promising candidate for sonogenetics.

TRP ankyrin 1 (TRPA1) is primarily expressed in sensory neurons and exhibits a broad reactivity to diverse stimuli, such as electrophilic compounds, reactive oxygen species, and a range of plant-derived compounds. A recent study demonstrated that TRPA1 also exhibits sensitivity to ultrasound.^84^ In the presence of low-intensity pulsed ultrasound (LIPUS), TRPA1 mediates the calcium influx in astrocytes, which in turn prompts the release of gliotransmitters like glutamate via the Best1 channel, thereby initiating action potentials in peripheral neurons.^85^ Notably, endogenous expression of *Homo sapiens* TRPA1 (hsTRPA1) in the brain is virtually undetectable.^86^ Hence, the introduction of exogenous hsTRPA1 in neurons could render them responsive to ultrasound stimulation, offering enhanced spatial resolution and precise targeting of specific brain regions. Experiments have shown that hsTRPA1 significantly amplifies the ultrasound-induced calcium influx and membrane currents in primary neurons. Furthermore, unilateral overexpression of hsTRPA1 in layer V motor cortex neurons of mice was found to elevate c-Fos expression and stimulate activity in the contralateral limb. Moreover, hsTRPA1 has been identified as a sensor for various perturbations, such as membrane stretch forces, lipid bilayer alterations, and cholesterol molecular interactions. Additionally, the association between actin and membrane cholesterol, coupled with their relationship to the C-terminus of hsTRPA1, can augment its ultrasound sensitivity and ion permeability. This potentiation of sensitivity could provide a beneficial advantage in the application of hsTRPA1 and its variants for noninvasive, cross-species neuronal and cellular regulation.

Several TRP channels, which have been identified to be expressed by neurons, may constitute the molecular foundation for the observed neural and behavioral responses to ultrasound stimulation.^64^ Notably, cortical neurons displayed heightened ultrasound sensitivity, even at reduced pulse intensities and durations, upon overexpression of TRP canonical 1, TRP polycystin 2, and TRP melastatin 4. Particularly in the case of TRP melastatin 4, the kinetics of the response were significantly accelerated.^87^ These findings suggest that these ion channels could play a critical role in ultrasound neuromodulation, potentially informing the development of *in vivo* sonogenetic strategies. Future research should explore the specificity of these proteins across different brain regions and neuronal subpopulations to refine the choice of these proteins for sonogenetic applications.

### Piezo

Piezo proteins, comprising Piezo1 and Piezo2 in vertebrates, are large membrane proteins structured by three monomers, which resemble three-lobed propellers. These proteins have the distinctive feature of crossing the membrane up to 114 times, designating them as the largest class of transmembrane proteins.^88^, ^89^, ^90^ The first 36 transmembrane regions of the monomers contain nine repetitive units, with one unit corresponding to each set of four transmembrane regions. The final two transmembrane segments are postulated to form a pore channel. These proteins possess the unique property of activation by pressure. Upon stimulation, they open, permitting cationic ions to cross the membrane. This characteristic enables Piezo proteins to facilitate cellular mechanotransduction, which assists in adaptation to the microenvironment.^91^ Both Piezo1 and Piezo2 are essential for mechanically activated currents in various cells. Overexpression of Piezo proteins in mammalian cells can induce a 17- to 300-fold augmentation in mechanically activated currents. Piezo1 exhibits selective conduction of cations such as Na^+^, K^+^, and Ca^2+^, whereas Piezo2 conducts ions non-selectively. These proteins are instrumental in transducing ultrasound-related mechanical signals and initiating downstream signaling processes. Upon exposure to LIPUS, both dental pulp stem cells and periodontal ligament stem cells have demonstrated increased expression of Piezo1 and Piezo2 proteins.^92^ Furthermore, the proliferation of dental pulp stem cells via LIPUS treatment was found to significantly decrease upon inhibition of Piezo-mediated ERK1/2 MAPK signaling.

Piezo1, observed in human brain-derived neural stem/progenitor cells, plays a critical role in neurogenesis and astrogliogenesis. The channel is responsive to ultrasound, which activates both heterologous and endogenous Piezo1. This activation triggers a calcium influx, enhancing nuclear c-Fos expression in primary neurons.^93^ It has also been proved that Piezo1 knockout mice displayed diminished limb movements and reduced EMG response, as well as decreased calcium signaling and c-Fos expression under particular ultrasonic stimulation.^94^ By specifically targeting the extracellular region of Piezo1 in nerve cells, Piezo1-targeted microbubbles can substantially decrease the ultrasound energy requirement needed for channel opening.^95^ Importantly, the ultrasound exposure dose does not result in microbubble disruption or significant temperature increase, implying a minimal involvement of cavitation and heating effects, thereby ensuring safety in ultrasound neuromodulation applications.

On the other hand, Piezo2 has a key function within the somatosensory system.^96^ Prominently expressed in the dorsal root ganglion, it contributes to the perception of light touch and proprioception. FUS is capable of exciting action potentials in peripheral neurons, including myelinated A fibers and unmyelinated C fibers, which are Piezo2-dependent.^97^ Piezo2 is also detected at the apical surface of the cuticular plate of cochlear outer hair cells and is especially sensitive to ultrasonic frequencies. In the absence of Piezo2, mice exhibited impaired ultrasonic hearing and reduced associative learning ability.^98^ As such, Piezo2, due to its heightened sensitivity to ultrasonic stimuli, may hold potential in sonogenetic applications.

### Two-pore domain potassium (K2P) family

The K2P family, a classification of potassium channels present in the mammalian nervous system and endothelial cells, plays a pivotal role in regulating neuroprotection, pain, and depression.^99^, ^100^, ^101^ These channels, also known as two-pore potassium channels, feature a structural composition comprising two functional domains, four transmembrane domains, and an extracellular cap. This combination operates in unison to stabilize the potentials of both excitable and non-excitable cell membranes, thereby modulating cellular membrane excitability.^102^ The K2P family contains 15 distinct members, subdivided into six subfamilies. Notably, TWIK-related potassium channel-1, TWIK-related potassium channel-2, and TWIK-related arachidonic acid-activated K^+^ channel (TRAAK) constitute mechanosensitive channels within the K2P family. In *Xenopus* oocytes expressing any of these channels, the application of ultrasound induces potent and sustained transmembrane currents, with the channels' activation considerably influenced by the concentration of extracellular K^+^ ions.^38^^,^^103^

Among these, TRAAK channel activation is contingent upon the tension within the lipid bilayer, enabling gating and cellular activity control via mechanosensitivity.^104^^,^^105^ TRAAK is specifically localized at the Ranvier nodes, key sites for action potential propagation in myelinated axons, and is implicated in the “leak” K^+^ current.^106^ Interestingly, the ultrasonic energy applied is transduced to TRAAK through the cell membrane without the involvement of other cellular components.^107^ Low-power stimulation prompts a robust activation of TRAAK with kinetics that can be up to 20 times faster. Owing to its low resting open probability coupled with its relatively high conductance properties, TRAAK is identified as a promising ultrasound-sensitive protein for the field of sonogenetics.

### Prestin

Prestin (SLC26A5) is a protein within the SLC26 anion transporter family. It is predominantly located in the outer hair cells of the cochlea and plays a vital role in high-frequency hearing by driving the electromotility of outer hair cells, functioning as an electromechanical transducer.^108^^,^^109^ This protein can detect alterations in membrane potential and, in response, can either elongate or contract along its axis to modify the membrane surface area, thereby facilitating signal amplification.^110^^,^^111^ The structure of Prestin features both an N-terminal domain and a C-terminal STAS domain, which stay on the cytoplasmic side of the cell, forming a dimerized “base”. Two transmembrane domains extend from this base, penetrating the cell membrane where they bind to chloride ions.^112^ Surrounding the Prestin transmembrane region, over 100 lipid molecules are found, along with a pair of cholesterol molecules located between the two transmembrane domains.^113^ The electromotility of Prestin is modulated by multiple factors, including intracellular anions (particularly chloride ions), cell membrane thickness, membrane tension, and cholesterol.^114^

Beyond its role in hearing, Prestin is also implicated in the process of echolocation in certain mammals.^115^ Several parallel amino acid substitutions in Prestin, such as N7T and N308S, are hypothesized to contribute to adaptive ultrasound hearing based on variances noted between the amino acid residues of echolocating and non-echolocating species.^116^^,^^117^ An engineered version of mouse Prestin, with two such substitutions (N7T, N308S), displayed an approximately 11-fold increase in sensitivity to low-frequency and low-energy ultrasound.^118^^,^^119^ This modified form of Prestin could potentially serve as an ultrasound-sensitive protein, enabling mammalian cells to respond to ultrasound stimulation.

The presence of the endogenous mechanically-sensitive ion channel can restrict the range of available sound pressure. As a result, it is crucial to enhance the sensitivity of the sonogenetic mediators and utilize short ultrasound pulse sequences at the lowest effective sound pressure to reduce non-specific effects. The ideal protein for sonogenetic application should possess the following characteristics: (i) a simple and uncomplicated structure that facilitates engineered expression within target cells; (ii) limited expression within target tissues to reduce the threshold for ultrasound stimulation; (iii) a distinct functional framework that allows for modification to enhance ultrasound sensitivity; and (iv) a well-defined understanding of its biological function when stimulated by ultrasound, thus promoting the desired cellular response.

### Ultrasound parameters in sonogenetics

**Figure 3:**
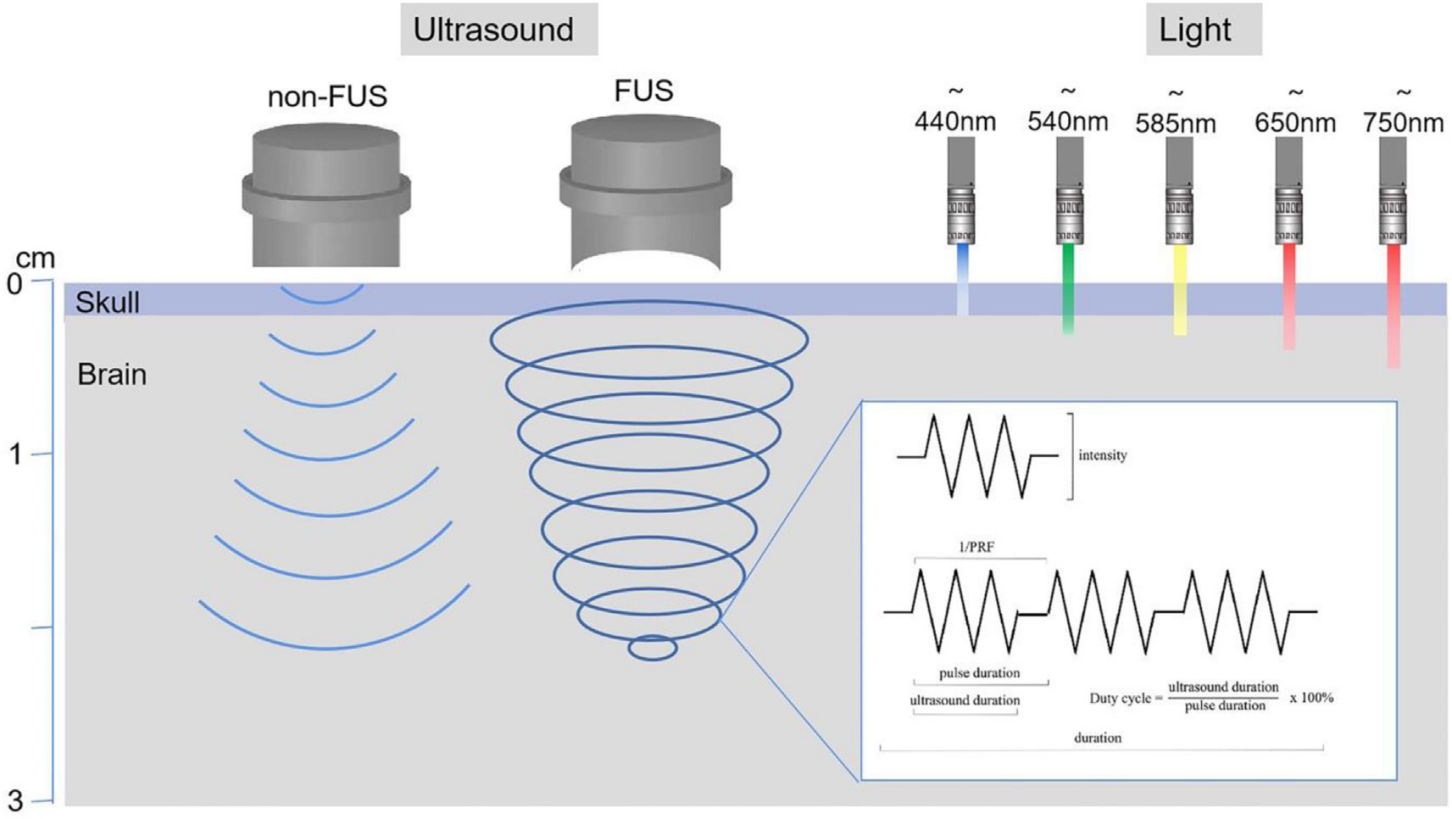
Ultrasound parameters.

Currently, the tissue-penetrating capacity of light waves used in optogenetics is restricted. Specifically, even far-infrared waves can only penetrate brain tissue to a depth of less than 1 cm. Conversely, ultrasound waves demonstrate considerably lower scattering and attenuation within tissues. At an ultrasound frequency of 15 MHz, imaging of the brain up to a depth of 2 cm with a resolution of 100 μm can be achieved. Existing research has shown that different ultrasound parameters can lead to varying biological effects, particularly regarding ultrasound-sensitive proteins. Therefore, the selection of ultrasound parameters in sonogenetics is pivotal in generating the desired biological outcomes. A thorough understanding of parameters such as intensity, frequency, duration, duty cycle, and pulse repetition frequency is essential, as depicted in Figure 3.

## Basic ultrasound parameters

### Intensity

The intensity of ultrasound refers to the acoustic energy it produces, usually quantified as the average intensity per pulse (termed spatial-peak pulse-average, or ISPPA) and the overall time-averaged intensity (referred to as spatial-peak temporal-average, ISPTA). Ultrasound intensities can be grouped into three categories: high (100–10,000 W/cm^2^), medium, and low (<3 W/cm^2^).^120^^,^^121^ High-intensity focused ultrasound primarily serves to induce hyperthermia for ablative procedures. Conversely, low-intensity focused ultrasound (LIFU) tends to generate mechanical or slight thermal effects, thereby producing biological modulatory impacts without significant accumulation of thermal energy.^122^, ^123^, ^124^ LIFU has been found to safely modulate cellular responses without causing damage, making it particularly useful in sonogenetic applications.

The biological impacts of ultrasound are strongly contingent on the intensity level. The response of neurons to LIFU can be both excitatory and inhibitory.^125^^,^^126^ Research has indicated that LIFU can enhance neuron excitability, stimulate intact brain circuits, and bolster the expression of endogenous neurotrophic factors. However, escalating intensity levels may depress synaptic transmission, induce tissue homogenization, instigate protein denaturation, and lead to DNA breakage.^127^ A key factor is the thickness of the human skull, which can attenuate nearly 76% of the incident FUS intensity during the transcranial process. One study found that the acoustic ISPPA at the targeted cortex is about 0.7 ± 0.5 W/cm^2^, based on 3D rendering and a coronal section of volumetric MRI data, with a transcranial FUS ISPPA of approximately 3 W/cm^2^.^128^ Therefore, understanding the variance in energy attenuation across tissues of different compositions and thicknesses is essential when defining ultrasonic parameters.

Acoustic pressure is a pivotal mechanical parameter indicative of ultrasound intensity and representative of the ultrasonic energy emitted. In the context of FUS, focal points are generated within deep tissues, thereby creating amplified biological impacts due to increased sound pressure and stimulation intensity. The effects of different acoustic pressures on neural cells are highly specific, which is particularly crucial in the domain of neuromodulation research. LIFU with an acoustic pressure exceeding 0.5 MPa has the potential to activate spinal cord neurocircuits and induce contraction in the soleus muscle. However, spinal cord injuries may arise with an excessive acoustic pressure of up to 3.0 MPa.^129^ Consequently, it is vital to regulate the acoustic pressure stringently within a reasonable range to prevent neural damage when utilizing LIFU to stimulate the central nervous system. The occurrence of microbubble cavitation under ultrasound is also contingent on acoustic pressure, distinguishing stable cavitation at low pressure from abrupt collapse at high pressure.^130^, ^131^, ^132^ Inertial cavitation transpires when the peak negative pressure surpasses a certain threshold, the value of which depends on the concentration of a solution or the amount of gas in the tissue, potentially leading to cell membrane damage.^133^

### Fundamental frequency

The fundamental frequency, defined as the rate of ultrasound oscillation within a set time frame, significantly impacts the utilization scope of ultrasound technology. High-frequency ultrasound (1–20 MHz) is predominantly employed in diagnostic imaging, while medium frequency (0.7–3 MHz) is customarily used for therapeutic purposes. In contrast, low-frequency ultrasound (20–200 kHz) is frequently employed in industrial contexts.^134^ The frequency governs both the tissue penetration depth and the spatial resolution. From a theoretical perspective, given that frequency and wavelength are inversely related, higher frequencies yield superior spatial resolution. However, it is important to note that as the frequency increases, tissue penetration is concurrently diminished. This escalation in frequency causes energy to attenuate as it is transmitted through the tissue, leading to a substantial generation of heat that disperses and damages the surrounding tissue. For this reason, it is crucial to calibrate the frequency range to amplify the efficiency of the ultrasound. This is because energy attenuation and conversion can undermine the overall effectiveness of ultrasound.^135^ In neuromodulation research, achieving the same level of efficiency with higher frequencies necessitates a more significant spatial peak intensity.^136^^,^^137^ Notably, a frequency exceeding 2.25 MHz can generate a focal spot size under a millimeter.^138^ Therefore, the lower frequencies may offer heightened efficiency and safety for *in vivo* applications. Enhancing the ultrasound sensitivity of the target cells represents a potential strategy for decreasing frequency.

### Duration, duty cycle, and pulse repetition frequency (PRF)

The term duration describes the total period from the inception of the initial pulse to the termination of the final pulse. Investigations utilizing a rat model have demonstrated that shorter pulse durations when applied to the motor area of the brain through ultrasound, induce neural excitation. Conversely, prolonged durations activate inhibitory neural circuits, thereby increasing the threshold.^139^ Given a steady ultrasound frequency and intensity, the effects magnify with duration extension. However, excessive pulse duration could induce a temperature surge, potentially causing local tissue damage. Hence, determining an optimal pulse duration necessitates a balance between amplifying stimulation and curtailing tissue damage.

Ultrasound can be transmitted in two forms: a continuous, uninterrupted flow referred to as continuous wave and a disrupted flow called pulse-mode stimulation. Evidence suggests that a singular pulse of ultrasound is inadequate for modulating neuronal excitability; the repetition of the singular pulse group is required to construct a comprehensive stimulus sequence. While both the continuous wave and pulse modes can initiate neural activation, the pulse mode is demonstrably more efficient. Moreover, an optimal duty cycle can be identified that minimizes the threshold for neural activation.

PRF, quantified in Hertz, signifies the quantity of pulses delivered per second. PRF can be computed as the inverse of the pulse repetition interval, which indicates the period between consecutive pulses. Contemporary research has uncovered a robust correlation between PRF levels and neuron modulation, revealing that high-PRF ultrasound (above 500 Hz) can effectively stimulate neural activity, as evidenced by evoked electroencephalogram.^139^ This discovery underscores the prospective utility of high-PRF ultrasound in neuroscience and neurology research.

### Response of ultrasound-sensitive proteins across different ultrasound parameters

The parameters of ultrasound, and their interplay, influence the impact of ultrasound stimulation in sonogenetics (Table 1). Experiments show that low-pressure ultrasound, with peak negative pressures ranging from 0.4 to 0.6 MPa, can selectively activate neurons that ectopically express TRP-4 when controlled via microbubbles-mediated regulation.^39^ Further, the MEC-4 mechanosensitive protein, an element of the DEG/ENaC/ASIC ion channel, has been proven necessary for the touch receptor neurons' response in *Caenorhabditis elegans* to FUS. Notably, these responses were reported with ultrasound stimuli having a peak negative pressure under 1 MPa, a frequency of 10 MHz, a duration of 200 ms, and a PRF of 1 kHz at a 50% duty cycle.^140^ *Caenorhabditis elegans* mutants lacking either TRP-4 or MEC-4 demonstrated that reintroducing TRP-4 into different neurons effectively restored significant response variations, including in AWC-chemosensory neurons at lower pressures (0.79 MPa) and ASH chemosensory neurons at higher pressures (>0.92 MPa).^141^ This implies that ultrasound-sensitive proteins within the same species may have divergent sensitivity levels to ultrasound-evoked stimuli.

**Table 1:** Ultrasound parameter settings and their corresponding effects.

Ultrasound-sensitive protein		Target	Ultrasound parameters	Microbubble	Effects	Reference
MscL	MscL-wild type	Proteoliposome model (*in vitro*)	ISPTA: 122–194 mW/cm^2^ inside the tank, 240–394 mW/cm^2^ inside the well; frequency: 0.5 MHz; duty cycle: 60%–100% (continuous wave); duration:10–20 min	No	The increase of calcein efflux under ultrasound modulation and membrane perturbation was induced when MscL channels were reconstituted into the proteoliposome model	57
	MscL-I92L	Rat hippocampal neurons (*in vitro*)	Peak negative pressure: 0.12–0.45 MPa; frequency: 29.92 MHz	No	In hippocampal neurons, the gain-of-function I92L mutation makes the ultrasound sensitive of MscL at 0.25 MPa, while the MscL-wild type cannot be activated even at 0.45 MPa	62
		Tumor cell lines (*in vitro* and *in vivo*)	Consistent ultrasound (6 MHz, 15 W) for 30 min	No	The apoptosis rate of the B16 cell line expressing MscL-I92L protein was increased by ultrasound	150
	MscL-G22S	Rat retinal ganglion cells (*in vivo*)	Duty cycle: 50%; PRF: 1 KHz; total duration: 10–200 ms Group 1: 0.5 MHz, 0.11–0.88 MPa, ISPPA 0.39–25.14 W/cm^2^ Group 2: 2.25 MHz, 0.3–0.6 MPa, ISPPA 29.2–83.12 W/cm^2^ Group 3: 15 MHz, 0.2–1.27 MPa, ISPPA 1.3–53.37 W/cm^2^	No	Ultrasound yields a spatial resolution of within 400 μm for stimulations at 15 MHz, the focal spot of our 15 MHz transducer being 276 μm wide. The MscL-G22S-transfected mice achieved a significantly higher success rate of visible-light perception	158
		Retinal pigment epithelial cells (*in vitro*)	0.071 MPa; frequency: 10 MHz; 25% duty cycle; PRF: 5 Hz; total duration: 10 s	Yes	Ultrasound-induced MscL channel opening is dependent on the functional linkage of the microbubbles with an intact actin cytoskeleton. The MscL-G22S mutant was activated at a lower threshold than the MscL-wild type	60
		Mouse primary neurons (*in vitro*)	0.15 MPa; center frequency: 500 kHz; duration: 300 ms; pulse width: 300 ms; duty cycle: 40%; PRF: 1 kHz	No	Expressing MscL-G22S significantly reduces the ultrasound intensity and provokes Ca^2+^ influx in mouse primary neurons	61
		Mouse cortex (*in vivo*)	0.05 MPa; duration: 300 ms; pulse width: 400 ms; duty cycle: 40%; center frequency: 500 kHz; PRF: 1 kHz	No	Ultrasound evokes muscular responses and targeted neurostimulation in the dorsomedial striatum region with MscL-G22S	
		Mouse primary motor cortex and dorsomedial striatum neurons (*in vivo*)	0.04–0.35 MPa; 0.5 MHz and 0.9 MHz; duration 300 ms; pulse width: 400–500 μs; PRF: 1 kHz; interval: 3 s	No	The MscL-G22S dorsal striatum regions showed stronger neuron responses than those of MscL-wild-type neurons. Ultrasonic stimulations on the MscL-G22S mice provided significant motor responses, modulated appetitive condition, and alleviated Parkinson's disease symptoms	59
TRP	TRP4	*C. elegans* (*in vivo*)	Peak negative pressure: <0.9 MPa; frequency: 2.25 MHz; duration: 10 ms	Yes	Neuron-specific misexpression of TRP-4 sensitizes neurons to ultrasound stimuli, resulting in behavioral outputs in *C. elegans*	39
			10 MHz frequency piezoelectric line-focused transducer	Yes	In both TRP-4 and MEC-4 mutants of *C. elegans*, TRP-4 expression in different neurons was able to partially rescue large reversals, AWC chemosensory neurons at lower pressures (0.79 MPa) and while ASH at higher pressures (>0.92 MPa)	141
	hsTRPA1	Mouse motor cortex (*in vivo*)	0.35–1.05 MPa, 7 MHz frequency, 1–100 ms duration, 7 MHz frequency	No	Unilateral expression of hsTRPA1 in mouse layer V motor cortical neurons leads to c-Fos expression and contralateral limb responses ultrasound delivered through an intact skull	83
	TRPV1	Mouse somatosensory cortex (*in vitro* and *in vivo*)	Peak negative pressure: 0.9–1.3 MPa; frequency: 1.5–1.7 MHz; 40% duty cycle; PRF: 10 Hz; duration: 15–30 s	No	FUS-induced thermal effect was sufficient to activate TRPV1-expressing cells *in vitro* and FUS stimulation of TRPV1-expressing neurons at the striatum repeatedly evoked locomotor behavior	32
		Mouse motor cortex (*in vivo*)	Peak negative pressure: 0.7 MPa and 1.1 MPa; frequency: 1.5 MHz; 40% duty cycle; PRF: 10 Hz; duration: 15 s	No	FUS-induced thermal effect was sufficient to activate TRPV1-expressing cells *in vivo*. FUS stimulation of TRPV1-expressing neurons in the motor cortex significantly induced rotational behavior	128
Piezo	Piezo1	Hippocampal neural cells (*in vitro*)	0.11–0.17 MPa; frequency: 2 MHz; duration: 300 ms	Yes	Piezo1-targeted microbubbles with hippocampal neural cells can enhance the effect of ultrasound stimulation	94
		Pancreatic cancer cells BxPC3 cells (*in vitro* and *in vivo*)	ISPPA: 1 W/cm^2^; duration: 10 min; 50% duty cycle	Yes	Piezo1 is activated by ultrasound with microbubbles and mediates calcium influx to induce apoptosis of pancreatic cancer cells	149
		T cells (*in vitro*)	0.6 MPa; frequency: 2 MHz; PRF: 100 Hz; 1% duty; duration: 10 min	Yes	Calcium influx occurred in T cells genetically engineered with Piezo1 and activated transcriptional activation for the CAR expression through the nuclear factor of activated T cell response element	143
Prestin	MPrestin (N7T N308S)	Primary neural cells (*in vitro*) and deep regions of mouse brains (*in vivo*)	0.5 MPa; 0.5 MHz; PRF: 10 Hz; 2000 cycles; duration: 3 s	No	An ultrasound pulse of low frequency and low pressure efficiently evoked cellular calcium responses after transfecting with prestin (N7T, N308S) and noninvasively stimulated target neurons expressing prestin (N7T, N308S) in deep regions of mouse brains	117
		Mouse deep brain regions (*in vivo*)	Ultrasound-induced blood-brain barrier disruption (0.3–0.7 MPa; frequency: 1 MHz; duration: 1 min; PRF: 1 Hz) combined with activation of the Prestin (N7T, N308S) neurons (0.5 MPa; frequency: 0.5 MHz; duration: 3 min; PRF: 10 Hz)	Yes	Prestin expression with pPrestin-microbubbles was the deepest two days after 1 MHz ultrasound stimulation. Prestin-expressing cells were 6-fold activated more than that in the cells without prestin expression with 0.5 MHz ultrasound stimulation	144
Heat shock protein		T cells (*in vitro* and *in vivo*)	The MRI-guided FUS system is composed of a 1.5 MHz 8-element annular array transducer; the tumor tissues received two pulses of 5 min FUS stimulation at 43 °C	No	The CAR-T cells in tumors can be reversibly controlled by the heat generated by short pulses of focused ultrasound via a CAR cassette controlled by a promoter for the heat-shock protein	151

The specificity of ultrasound deep brain stimulation can be enhanced by expressing ultrasound-sensitive proteins from non-mammalian sources in targeted mammalian brain regions. The MscL protein, when expressed in human retinal pigment epithelial cells, can be activated by ultrasound actuation of integrin-anchored microbubbles using an acoustic pressure of 0.07 MPa, a frequency of 10 MHz, a 10-s duration, a 50-ms pulse duration, and a PRF of 5 Hz.^60^ The MscL-G22S mutant has shown a lower activation threshold compared with the wild-type MscL.^61^ Mouse primary neurons expressing MscL-G22S exhibited an increased Ca^2+^ influx in response to ultrasound stimulation employing 0.15 MPa, a center frequency of 500 kHz, a duration of 300 ms, a pulse width of 300 ms, a 40% duty cycle, and a PRF of 1 kHz. In a similar vein, MscL-G22S-expressing mouse cortices *in vivo* were responsive to ultrasound stimuli, resulting in heightened muscular responses at 0.05 MPa, a stimulation duration of 300 ms, pulse width of 400 ms, 40% duty cycle, a center frequency of 500 kHz, and a PRF of 1 kHz.^61^ Moreover, the I92L mutant, which carries a gain-of-function mutation, had a significantly lower activation threshold. Specifically, the I92L mutation rendered MscL sensitive to ultrasound at 0.25 MPa in the absence of microbubbles in hippocampal neurons, while the MscL-wild type remained unresponsive until 0.45 MPa.^62^ Therefore, while microbubbles amplify the response of MscL to ultrasound, the gain-of-function mutations in MscL offer an alternative approach to circumvent the constraints of *in vivo* microbubble application.

Studies have revealed that hsTRPA1 is superior to other channel proteins, including MscL, Prestin, TRPV1, and Piezo, in response to ultrasound frequencies of 1 MHz, 2 MHz, and 7 MHz, underscoring its broad sensitivity to a variety of ultrasound stimuli.^85^ Furthermore, hsTRPA1 is capable of imparting ultrasound sensitivity to mammalian cells at an optimal frequency of 7 MHz, presumably concentrating this effect to a compact volume of 107 μm^3^ without inducing cavitation. This characteristic enables the selective activation of motor cortical neurons in hsTRPA1-expressing mice through ultrasound pulses (duration: 1–100 ms; frequency: 7 MHz; pressure: 0.35–1.05 MPa). In contrast, TRPV1, selectively expressed in mammalian neurons, can be activated with high temporal precision by FUS (peak negative pressure: 0.9–1.3 MPa; frequency: 1.5–1.7 MHz; 40% duty cycle; PRF: 10 Hz; duration: 15–30 s), resulting in a tissue temperature elevation to roughly 42 °C.^33^ Given their distinct ultrasound sensitivities, the selection between TRPV1 and hsTRPA1 channels primarily depends on the target cell and the specific research objective. For instance, TRPV1, activated by heat, is suitable for use in tumor tissue but requires cautious use in neural tissue due to the potential for neurotoxicity from temperature increases.

Piezo1, which is endogenously expressed in various cell types, can potentially be activated by ultrasound. In neurons, an ultrasound at 43 MHz and intensities of either 50 or 90 W/cm^2^ can alter neuronal electrical activity and activate Piezo1.^142^ Furthermore, ultrasound stimulation of dental pulp stem cells and periodontal ligament stem cells necessitates intensities of 250 mW/cm^2^ and 750 mW/cm^2^, respectively.^92^ Consequently, the response threshold of Piezo1 to ultrasound varies among different cell types. In hippocampal neural cells, microbubbles targeting Piezo1 can amplify the effects of ultrasound stimulation at pressures of 0.11–0.17 MPa, a frequency of 2 MHz, and a duration of 300 ms. Furthermore, T cells genetically modified with Piezo1 can induce calcium influx, which triggers the transcriptional expression of the chimeric antigen receptor (CAR) via the Ca^2+^/nuclear factor of activated T cells response element when exposed to low-frequency 2 MHz ultrasound in the presence of microbubbles.^143^ Therefore, the use of Piezo1-targeted microbubbles may enhance the opening of channels in response to mechanical stress, thereby increasing ultrasound sensitivity.

To surmount the hindrance of low penetration depth that high-frequency ultrasound exhibits in *in vivo* applications, heterologous expression of mPrestin (N7T, N308S) has demonstrated its capability to manipulate mammalian cell activities via low-frequency and low-pressure ultrasound. Through *in vitro* examinations, FUS (with a frequency of 0.5 MHz, and a peak negative pressure of 0.5 MPa) has proven capable of inducing robust calcium responses in cells transfected with Prestin (N7T, N308S). Similarly, neurons expressing Prestin (N7T, N308S) within deep regions of murine brains have been effectively and noninvasively stimulated.^118^ For the implementation of an efficient sonogenetic system that allows remote mammalian brain control, a strategy employing ultrasound-induced disruption of the blood-brain barrier (under conditions of a pressure of 0.3–0.7 MPa, a frequency of 1 MHz, a duration of 1 min, and a PRF of 1 Hz) in conjunction with low-frequency ultrasound stimulation (frequency: 0.5 MHz; pressure: 0.5 MPa; PRF: 10 Hz; duration: 3 s) of neurons expressing Prestin (N7T, N308S) has been employed for precise spatiotemporal neuromodulation.^144^

The intensity and frequency are paramount parameters that govern the biological effects of ultrasound, among several other factors. One crucial aim of sonogenetics is the reduction of ultrasound application intensity and frequency to enhance the tissue depth of ultrasound action and target cell response specificity. Equating biological effects can be achieved by adjusting the duration, duty cycle, and PRF settings, which in turn can influence the applied intensity and frequency. Nonetheless, establishing a universal standard for sonogenetics ultrasound parameters remains a formidable challenge due to variances in basic settings across different laboratories and equipment.

## Sonogenetic application

### Neuromodulation and nervous system disease

A multitude of techniques, such as electrophysiology, calcium influx monitoring, and optogenetics, offer unique ways to monitor neural activity selectively and understand neural circuits. While optogenetic neuromodulation presents high spatiotemporal resolution and cell type specificity, its application is hampered by restricted light penetration, particularly to deep brain structures.^145^ Conversely, sonogenetics, employing ultrasound waves, enables noninvasive deep penetration into brain tissue, targeting specific cells with high precision. Most sonogenetic neuromodulation studies have documented neural calcium influx and evoked action potentials. Electromyogram traces have been captured to record muscle responses consequent to transcranial stimulation of brain regions governed by sonogenetics, thereby demonstrating the capability of sonogenetics to manipulate muscle function through neurostimulation. To further enhance the application of sonogenetics, it is vital to conduct additional research exploring the underlying neural circuits and their functionality.

Deep brain stimulation, a technique involving surgical implantation of electrodes to activate specific neuron subsets, is predominantly employed in clinics for treating Parkinson's disease, epilepsy, and various other nervous system disorders.^146^ However, this procedure can potentially expose patients to risks of postoperative infection and subsequent side effects. Recent research has revealed that the long-term expression of mPrestin (N7T, N308S) in dopamine neurons could be realized following a single injection of an adeno-associated virus vector expressing mPrestin (N7T, N308S). Under repetitive ultrasound stimulation, a substantial dopaminergic enhancement was noted in a mouse model of Parkinson's disease.^147^ In Parkinson's disease mice, repeated ultrasound stimulation on neurons in the ventral tegmental area that express MscL-G22S can effectively target the activation of subthalamic nucleus neurons and alleviate dyskinesia in these mice.^59^ Given the safety of localized virus vector injection, it may be more feasible to permeabilize the blood-brain barrier using ultrasound in conjunction with plasmid transfection.^144^ These results suggest that sonogenetics holds immense clinical promise for treating neurodegenerative diseases. To optimize its application, the combination of multiple biological effects of ultrasound should be contemplated. However, the efficacy of sonogenetic therapy in treating neurodegenerative diseases remains to be verified after long-term treatment.

### Oncological therapies

Traditional methods of oncological treatment such as chemotherapy or radiotherapy often lack precision, inducing cell death in healthy cells alongside malignant ones. This non-specificity commonly leads to systemic side effects including nausea, alopecia, and decreased immunity. However, recent research has illustrated that low-intensity ultrasound can evoke an immune response specific to cancer cells, and enable the targeted delivery of chemotherapy agents. Advancements in sonogenetics have introduced therapeutic approaches that offer increased safety and precision, thereby inducing apoptosis in tumor cells and effectively controlling tumor immunotherapy.

Through the ultrasound-mediated activation of Piezo1, a cycle of Ca^2+^ influx is instigated which relates to microtubule disruption, thereby inducing calcium-dependent apoptosis.^148^ Research has indicated that Piezo1-mediated Ca^2+^ influx can be induced by ultrasound in pancreatic cancer cells, given the presence of microbubbles.^149^ Moreover, cationic nanoliposomes have been shown to be effective carriers for MscL I92L expression plasmids, capable of delivering them to tumor cells *in vivo*. This facilitates tumor cell death via an overload of Ca^2+^ influx triggered by ultrasound.^150^ These studies underscore the potential of sonogenetics to mediate apoptosis in tumor cells through Ca^2+^ influx, presenting a noninvasive approach to cancer therapy.

Immunotherapy, particularly involving CAR-expressing T cells, shows significant promise in cancer treatment. However, challenges exist in the form of non-specific targeting of CAR-T cells against normal and non-malignant tissues. There is a pressing requirement for precise spatiotemporal control over the genetic activation of these CAR-T cells. Ultrasonic mechanical effects, augmented by microbubbles, can accurately control CAR expression in T cells via engineered Piezo1 activation.^143^ Transient heat stimulation from short-pulsed FUS can induce CAR expression, regulated by a promoter for the heat-shock protein.^151^ Given that solid tumors frequently share antigens with normal tissues, utilizing ultrasound to regulate engineered T cells to express CAR at suitable times and locations for the detection and elimination of tumor cells could be an alternative strategy.

### Ophthalmic therapy

The therapeutic potential of optogenetics in ameliorating degenerative ocular conditions such as glaucoma and retinitis pigmentosa, is supported by an expanding body of research.^152^ Evidently, optogenetic methodologies can effectuate partial restoration of visual functionality at the retinal and visual cortex strata.^153^, ^154^, ^155^ Notably, the intrinsic penetrative capability of ultrasound extends to deep tissues, inclusive of the visual cortex in non-human primates.^156^ Utilizing 15 MHz ultrasound waves, neurons in the visual cortex expressing MscL-G22S can yield millisecond temporal resolution and 100 μm spatial resolutions.^20^^,^^157^ The latest study has also demonstrated that ultrasound can active retinal ganglion cells or visual cortex expressing MscL-G22S in milliseconds with a spatial resolution of at least 400 μm, which could provide great hope for the development of high-resolution visual restoration.^158^ Consequently, sonogenetic interventions present a prospective avenue for the revitalization of vision in patients suffering optic atrophy in glaucoma or diabetic retinopathy contexts. Nevertheless, the efficacy, safety, and optimization of parameter settings necessitate further empirical investigation.

### Stem cell research and therapy

LIPUS has demonstrated an influential effect on the proliferation, differentiation, and migration of diverse stem/progenitor cells, including bone marrow mesenchymal stem cells, adipose tissue-derived stem cells, spermatogonial stem cells, neural stem cells, and hematopoietic stem cells.^159^, ^160^, ^161^, ^162^, ^163^, ^164^, ^165^, ^166^, ^167^ Empirical evidence suggests that LIPUS stimulates responses in a variety of stem cell signaling pathways, namely YAP, RhoA/ROCK, Piezo, angiotensin, TGF-β1, MAPK, ERK, and protein kinase B (Akt). These responses to LIPUS vary according to the energy level applied and the origin of the cells. Stem cells display an inherent sensitivity to ultrasound waves, attributable to the presence of mechanosensitive membrane channels on their surface. Specifically, the regulation of ERK1/2 MAPK signaling via Piezo1 significantly contributed to LIPUS-induced cell proliferation in dental stem cells.^90^ Further, LIPUS has been shown to facilitate endothelial differentiation and microvascular formation in periodontal ligament stem cells through the activation of Piezo1 expression.^168^ LIPUS has also been found to enhance VEGF secretion by adipose tissue-derived stem cells, contributing to its therapeutic efficacy in treating diabetic erectile dysfunction through the activation of the Piezo-ERK-VEGF pathway.^169^ The presence of these mechanosensitive channels opens the avenue for the utilization of sonogenetics in stem cells, which aids in fine-tuning the effects of LIPUS on stem cells for the treatment of conditions such as fractures and osteoporosis.

The integration of stem cell technology and sonogenetics offers a promising approach for finely-tuned spatial and temporal manipulation of stem cell behavior. Recent studies have demonstrated that an innovative optogenetic system, centered on human-induced pluripotent stem cells, facilitates the steering of TGF-b-mediated mesenchymal differentiation. Furthermore, human-induced pluripotent stem cells can function as a cellular model for the optogenetic regulation of insulin secretion, implying prospective therapeutic applications in hyperglycemic disorders.^170^^,^^171^ In considering future developments, it is crucial to acknowledge that optogenetic stimulation in deep tissue presents certain obstacles due to light absorption and scattering. Thus, sonogenetic stimulation may emerge as a more appropriate method for controlling diverse stem cell types, especially within deep tissue. Moreover, sonogenetics holds promise for the spatial and temporal control of biological activity of transplanted stem cells *in vivo*.

### Others

Sonogenetics has the potential to manipulate diverse cellular types and tissues through ultrasound technology. For example, ultrasound-sensitive proteins expressed by cardiomyocytes can be remotely activated via ultrasound waves, obviating the requirement for traditional pacemaker implantation. Moreover, sonogenetic tools enable the selective activation or inhibition of cardiomyocytes, and they can potentially serve as a defibrillatory mechanism to abate arrhythmia in patients with severe cardiac complications.

## Summary

Sonogenetics, a technique that combines genetic engineering and the unique biological impacts of ultrasound, has emerged as a precise approach for modulating biological activities in targeted tissues via ultrasound-sensitive proteins. This noninvasive technology, characterized by its exceptional spatiotemporal resolution, shows significant potential in diverse areas, including deep brain neuromodulation, tumor immunotherapy, and stem cell therapy. Despite its substantial potential, attracting considerable interest among researchers, sonogenetics is not without limitations. These encompass the requirements for specialized equipment and specific ultrasound parameters, as well as the means of ultrasound-sensitive protein expression. Further, it presents an inadequately defined regulatory mechanism. Unlike photosensitive proteins that react to specific light wavelengths, most ultrasound-sensitive proteins are mechanosensitive and lack specificity in ultrasound response.

The application of sonogenetics could be expanded by further investigations into the structural characteristics of ultrasound-sensitive proteins to enhance their sensitivity and by identifying proteins that inhibit or switch off ultrasound response activity. Research into ultrasound-sensitive proteins expressed in primates, or genetically engineered ultrasound-sensitive proteins with lower thresholds for cellular ultrasound response, will also offer valuable insights for future directions. Moreover, it is anticipated that the inherent qualities of deep ultrasound stimulation, when combined with sonogenetics, may provide effective regulation of myocardial or pancreatic tissue, potentially benefiting the treatment of heart disease and diabetes. Although this novel technology requires additional research to fully understand its therapeutic potential, sonogenetics is expected to make a substantial contribution to the biomedical field in the near future.

## Author contributions

JT studied the literature and drafted the manuscript under the supervision of KY. MF produced the figures. DW and LZ assisted in manuscript collation and review. KY as the corresponding author conceived the review and provided critical input. All authors contributed to the article and approved the submitted version.

## Conflict of interests

The authors declare that there is no conflict of interests.

## Funding

This work was supported by the 10.13039/501100001809National Natural Science Foundation of China (No. 81771845) and the Chongqing Science and Technology Committee, Chongqing, China (No. CSTB2022NSCQ-MSX0812).
